# Parametric analysis of electromagnetic wave interactions with layered biological tissues for varying frequency, polarization, and fat thickness

**DOI:** 10.1038/s41598-025-33460-2

**Published:** 2025-12-26

**Authors:** Akram Gasmelseed

**Affiliations:** Department of Health Informatics, College of Applied Medical Sciences, Qassim University, Buraydah, 52571 Saudi Arabia

**Keywords:** Electromagnetic dosimetry, Layered tissues, TE/TM oblique incidence, Pennes bioheat, Power reflection, Temperature rise, Biophysics, Engineering, Materials science, Physics

## Abstract

Electromagnetic wave interaction with biological tissue is frequency-, angle-, and polarization-dependent, influencing both dosimetric parameters and resultant thermal effects. This work presents a comprehensive analysis across the major ISM bands (433, 915, 2450, and 5800 MHz) for transverse electric (TE) and transverse magnetic (TM) polarizations incident on a three-layer tissue model (skin–fat–muscle). A custom MATLAB code was developed to integrate the multilayer transmission line formalism, polarization-specific wave impedance modeling, Cole–Cole dielectric parameterization, and a finite difference method (FDM) solution of the Pennes bioheat equation. Simulations were performed for incident power density 50 W/$\hbox {m}^2$ and fat thicknesses from 0.005 m to 0.03 m, over incidence angles 0 °C– 80 °C. Throughout the manuscript, reflection is reported strictly as a *power* quantity $R=|\Gamma |^2$ rather than a field-amplitude coefficient. The thermal pipeline solves the steady-state Pennes equation in its direct $\Delta T$ form with consistent surface (Robin) and deep (Dirichlet) boundary conditions, and simulations are audited by an energy-conservation budget. Results indicate that while temperature increases remain below 0.4 °C at lower frequencies (433–915 MHz), significant superficial heating (up to 3.5 °C) occurs at 5.8 GHz due to reduced penetration depth, even at moderate exposure levels. The results demonstrate that subcutaneous fat acts as a low-loss impedance transformer whose thickness strongly modulates the balance between reflection and internal absorption, while polarization and angle primarily tune the detailed shape of angular reflection curves (including TM Brewster-like minima) at a given incident power. The analytical framework therefore complements voxel-based full-wave numerical models by providing fast, physically transparent trends across ISM bands that are directly relevant for preliminary assessment of wearable devices, implanted sensors, and compliance with radiofrequency safety limits.

## Introduction

The deposition of electromagnetic energy within biological tissues involves complicated dependencies on incident wave frequency, field strength, polarization characteristics, incidence angles, and tissue electrical properties^1^. Additionally, the thickness of the subcutaneous fat layer, as a major morphological variable, significantly impacts energy transfer and penetration depth^2,3^. Further, the continued development of internet of things (IoT) networks, 5G cellular technology, and improved medical wireless monitoring has created a far more complex electromagnetic backdrop in daily life. This reality emphasizes how crucial accurate radiofrequency exposure measurements are for humans, both to maintain public health standards and to ensure wearable wireless technologies deliver peak performance. International safety organizations including ICNIRP and the IEEE’s C95.1 committee have developed protocols using SAR metrics and temperature-based limits to prevent adverse health consequences^4,5^. High-fidelity three-dimensional computational phantoms generate precise dosimetric information, but they require intensive computational processing and commonly hide the fundamental physical processes controlling electromagnetic wave transmission in biological media^6^. One-dimensional (1D) analytical models, although idealized representations of the human anatomy, offer a computationally efficient approach to systematically investigate the impact of various parameters and understand the underlying physics of wave reflection, transmission, and interference in layered structures^7–9^. Canonical planar stacks have therefore been used for more than four decades to identify worst-case absorption conditions, to benchmark numerical solvers, and to isolate the influence of individual parameters such as layer permittivity, thickness, and incidence angle before moving to anatomically detailed simulations^8,10^. Previous research has used 1D modeling approaches to study specific aspects of RF exposure. Studies have consistently demonstrated that frequency plays a crucial role in how deeply signals penetrate materials: the higher the frequency, the more energy is deposited superficially^11,12^. However, the majority of available studies emphasize normal incidence or a limited set of oblique angles, and often treat the incident field as effectively unpolarized, so that systematic differences between TE and TM polarizations under realistic exposure conditions remain underexplored^13–15^. Although researchers understand that fat layers serve as impedance matchers between skin and muscle tissue, comprehensive systematic analysis across wide frequency bands, various angles, and different thicknesses has been uncommon. This study addresses these gaps by providing an extensive parametric investigation of electromagnetic wave behavior in a simplified skin–fat–muscle tissue model. The planar model does not aim to reproduce the fine anatomical details of a specific body site but rather to expose dominant trends and worst-case combinations of frequency, polarization, and morphology in a controlled setting. The present investigation examines four major Industrial, Scientific, and Medical (ISM) frequency bands (433 MHz, 915 MHz, 2.45 GHz, and 5.8 GHz), systematically varying fat thickness, wave incidence angles, and polarization types (TE and TM). Using the Transfer Matrix Method (TMM) combined with the Pennes bioheat equation, the study calculates reflection coefficients, SAR distributions, and the resulting temperature increases. The TE/TM decomposition is adopted because any arbitrary plane wave can be represented as a superposition of these two fundamental polarizations and, even in non-magnetic biological media, the associated boundary conditions at dielectric interfaces lead to markedly different reflection and transmission behavior – in particular, to the existence of a Brewster angle for TM but not for TE waves^16^. The contributions of this work include clarifying how fat tissue acts as a dynamic impedance transformer and supplying detailed measurements of polarization effects on wave behavior during oblique incidence, providing basic understanding of how exposure varies across stratified biological tissues. In addition, the coupling between depth-dependent SAR and steady-state temperature rise is quantified so that the relative importance of dielectric and thermal parameters (such as conductivity, perfusion, and thermal conductivity) can be assessed in a unified framework across the complete ISM band.

Comprehensive electromagnetic exposure evaluation in the 433–5800 MHz ISM spectrum depends on sophisticated simulation techniques to model field penetration and absorption mechanisms in living tissues for various polarization states and incidence configurations. Present dosimetric modeling methodologies commonly examine individual frequencies under perpendicular incidence conditions, constraining their utility for detailed safety validation and clinical parameter enhancement. The coupling of EM fields with biological structures produces frequency-dependent dielectric behavior, impedance boundary mismatches, and complex thermal responses that can be modeled using the Pennes bioheat transfer theory. Current literature lacks systematic analysis coupling electromagnetic propagation with thermal modeling across the complete ISM spectrum for both TE and TM polarizations. At the same time, recent experimental studies on intra-body and on-body communication through adipose tissue and tissue-mimicking phantoms have demonstrated that fat can behave as a relatively low-loss propagation channel in the sub-6-GHz range^17–20^, underscoring the need for simple yet quantitative models that relate layer morphology to transmission, reflection, and temperature rise. This work addresses three significant shortcomings: (1) detailed multi-frequency examination (433, 915, 2450, 5800 MHz) quantifying frequency-related energy absorption variations, (2) thorough TE/TM polarization study revealing different reflection characteristics and SAR behaviors, and (3) incidence angle analysis (0 °C–80 °C) exploring Brewster angle impacts and peak absorption situations. The transmission line model employs characteristic matrix formulations together with four-pole Cole–Cole dielectric representations to describe skin, adipose, and muscle layer properties. Thermal analysis solves the steady-state Pennes equation using finite difference methods with convective surface boundaries. All numerical models apply an incident power density of 50 W/$\hbox {m}^2$ to provide equivalent conditions for ISM frequency band comparison. The analysis indicates frequency-dependent SAR concentration levels between 0.3 and 2.1 W/kg, reflection variations of 15–40% depending on field polarization, and absorption peaks at tissue-specific Brewster angle orientations. The integrated electromagnetic–thermal framework thus provides a rapid screening tool that identifies how realistic variations in subcutaneous fat thickness (5–30 mm), polarization, and angle modify both dosimetric quantities and the ensuing steady-state temperature rise, in support of RF safety assessments and the preliminary design of wearable and implanted medical devices.

## Mathematical analysis

**Fig. 1:**
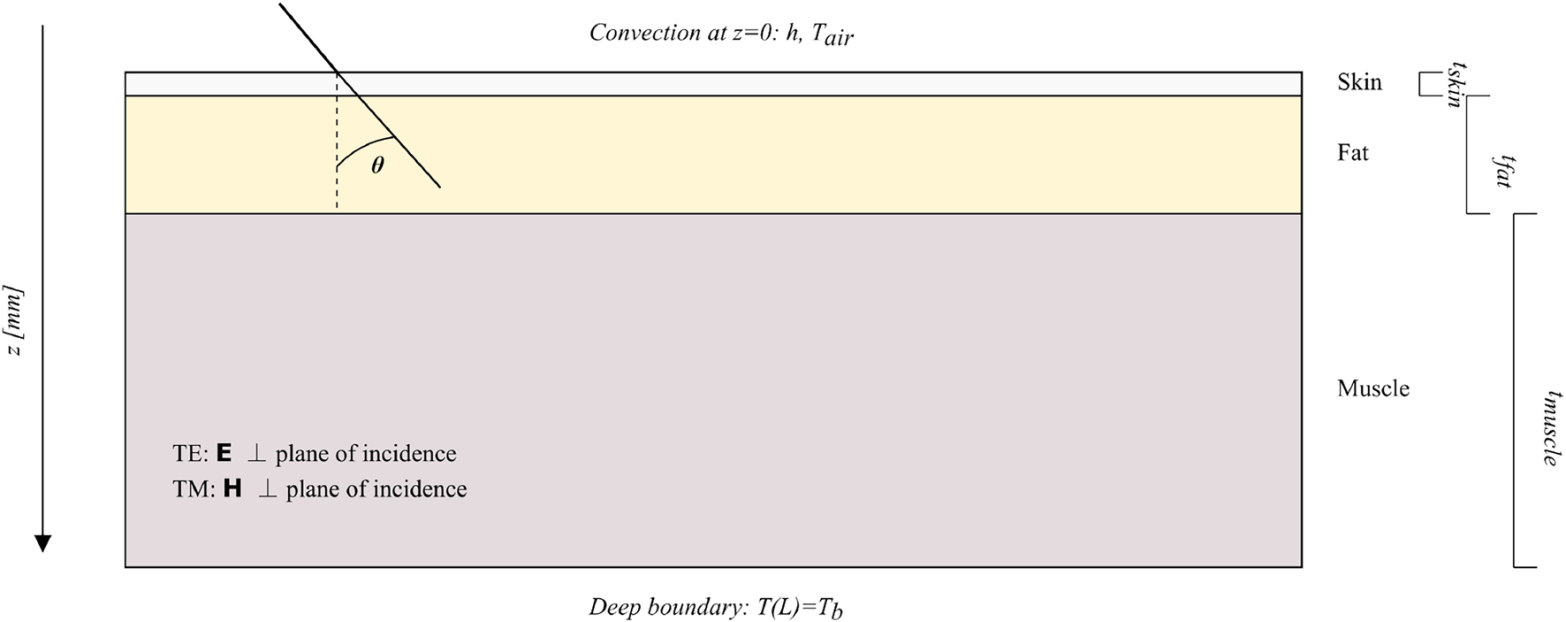
Three-layer tissue model showing skin, fat, and muscle layers with obliquely incident electromagnetic wave. The model demonstrates wave propagation through different tissue interfaces with varying dielectric properties. The schematic fixes the reference frame (positive *z* downward), TE/TM conventions, and the thermal boundary conditions: convection at $z{=}0$ with coefficient *h* and $T_{\textrm{air}}$, and a deep Dirichlet clamp $T(L){=}T_b$.

**Fig. 2:**
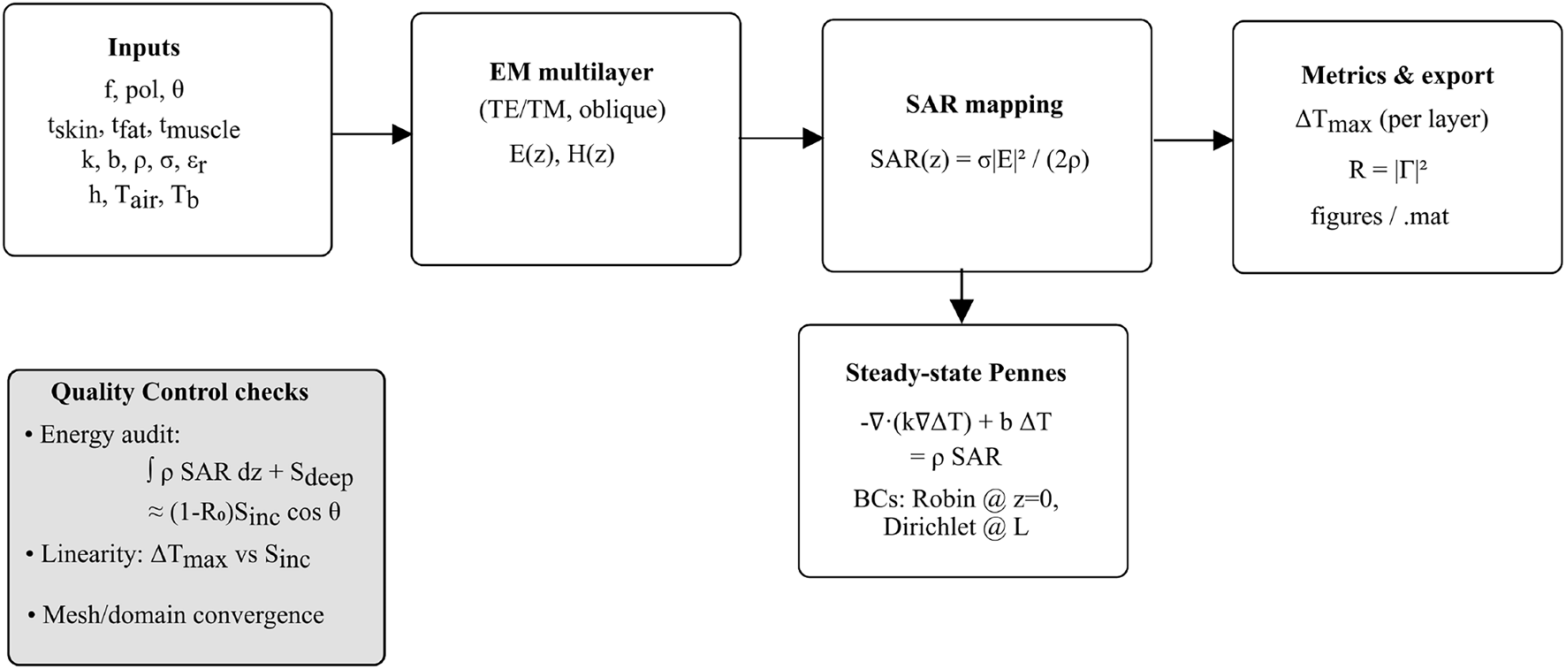
Modelling workflow and QC. Inputs (frequency, polarization, angle, layer thicknesses and properties) feed a multilayer EM solver to obtain *E*(*z*) and $\textrm{SAR}(z)$. The steady-state Pennes equation in direct form is then solved with a convective Robin boundary at $z{=}0$ and a deep Dirichlet condition to obtain $\Delta T(z)$ and per-layer $\Delta T_{\max }$. Each case is audited by an energy-conservation budget and checked for linearity and mesh/domain convergence prior to export.

A canonical three-layer planar model representing human tissue (skin, fat, muscle) was employed for this investigation (Fig. 1). The analysis involved a two-step approach: electromagnetic simulation to determine power deposition, followed by thermal simulation to calculate temperature elevation. A high-level workflow of the end-to-end pipeline and the quality-control (QC) steps is summarized in Fig. 2. This decomposition into an EM and a bioheat stage follows common practice in bioelectromagnetics^10,21–23^, allowing the same EM field solution to be reused under different thermal parameter sets and facilitating comparison with existing benchmark configurations.

### Tissue model and dielectric properties

The model assumes infinite planar layers. The skin layer thickness was fixed (e.g., 2 mm). The subcutaneous fat layer thickness was varied parametrically from 5 mm to 30 mm in 5 mm increments to represent inter-subject variability. The muscle layer was treated as semi-infinite, assuming negligible reflection from deeper tissues. The electromagnetic properties (permittivity $\varepsilon _r$ and conductivity $\sigma$) of the tissues were modeled with proven parametric dispersion approaches, including Cole–Cole models (Table 1), based on parameters from experimental studies^24^. The thermal properties (thermal conductivity, specific heat capacity, blood perfusion rate, and density) were assumed to be constant across the frequency range (Table 2), using standard literature values^10,21,25^. The chosen fat thickness range of 5–30 mm brackets the subcutaneous adipose layers typically represented in numerical head and trunk models used for dosimetry^2,3,12^, thereby spanning realistic morphologies from lean to overweight subjects while keeping the parameter space manageable. Within each layer, the material is treated as homogeneous and isotropic; finer heterogeneity (for example, fibrous structures within muscle) could in principle be captured by further subdividing the layer into multiple sublayers with different Cole–Cole parameter sets, but this added complexity was not required for the parametric trends targeted in the present work.

**Table 1:** Cole–Cole dielectric parameters for three-layer tissue model at body temperature (37 °C). Source: IT’IS Foundation database^25^. Parameters used for Cole–Cole dispersion modeling in the ISM frequency bands (433 MHz–5.8 GHz). Dashes (—) indicate parameters not specified for the respective tissue type.

Parameter	Skin (dry)	Fat (ave. infiltrated)	Muscle
$\varepsilon _{\infty }$	4.0	2.5	4.0
$\Delta \varepsilon _1$	32	9	50
$\tau _1$ (ps)	7.234	7.958	7.234
$\alpha _1$	–	0.20	0.10
$\Delta \varepsilon _2$	1100	35	7000
$\tau _2$ (ns)	32.48	15.92	353.7
$\alpha _2$	0.20	0.10	0.10
$\sigma _s$ (S/m)	0.0002	0.035	0.20
$\Delta \varepsilon _3$	–	$3.3 \times 10^4$	$1.2 \times 10^6$
$\tau _3$ ($\mu$s)	159.2	159.2	318.3
$\alpha _3$	0.20	0.05	0.10
$\Delta \varepsilon _4$	–	$1.0 \times 10^7$	$2.5 \times 10^7$
$\tau _4$ (ms)	15.92	15.92	2.274
$\alpha _4$	0.20	0.01	–

**Table 2:** Thermal properties and tissue thicknesses used in the three-layer analytical model.

Property	Skin	Fat	Muscle
Mass density $\rho _n$ (kg/$\hbox {m}^3$)	1109	911	1090.4
Specific heat $c_n$ (J/$\hbox {kg}^\circ$C)	3500	2500	3600
Thermal conductivity $\kappa _n$ (W/$\hbox {m}^\circ$C)	0.42	0.25	0.50
Blood perfusion $b_n$ (W/$\hbox {m}^3$ $\vphantom{0}^\circ$C)	9100	1700	2700
Metabolic heat $A_n$ (W/$\hbox {m}^3$)	1620	300	480
Tissue thickness (mm)	2	varies	30

Thermal parameters adapted from^21^ ^10^. Fat thickness is varied parametrically from 5–30 mm to investigate morphological effects on electromagnetic energy deposition and thermal response.

The tissue stack was assumed to be irradiated by a uniform plane wave with a defined polarization (TE or TM) and incident angle $\theta$ (varied from 0 °C to 80 °C). For analyzing electromagnetic wave propagation through the multi-layer system, the TMM was implemented^9,13^. TMM provides an analytical solution for the reflection and transmission coefficients at each interface by relating the electric and magnetic fields at the beginning and end of each homogeneous layer. Such multilayer transmission-line analogies have been widely adopted in bioelectromagnetics to interpret how the tissue stack behaves as an impedance transformer between free space and deeper tissues^8,9^, thereby offering a natural bridge between analytical understanding and full-wave numerical simulations.

### Electromagnetic analysis

The stack of tissue was assumed to be irradiated by an *x*-directed uniform plane wave with a defined polarization (TE or TM) and incident angle $\theta _i$, which varied from 0 °C to 80 °C. For a given layer *i*, the relationship between the tangential fields at the interfaces is given by the transfer matrix $M_i$ as,

1 $$\begin{aligned} M_i = \begin{pmatrix} \cos (\beta _i d_i) & jZ_i \sin (\beta _i d_i) \\ jY_i \sin (\beta _i d_i) & \cos (\beta _i d_i) \end{pmatrix} \end{aligned}$$

where $d_i$ is the tissue thickness, $\beta _i$ is the complex propagation constant, $Z_i$ is the wave impedance, and $Y_i$ is the wave admittance of layer *i*. The wave impedance ($Z_i$) depends on the polarization (TE or TM) and the angle of incidence ($\theta _i$) within the layer, determined by Snell’s law. For TE polarization, the electric field is perpendicular to the plane of incidence, whereas for TM polarization it lies within that plane; as a result, the effective impedance scales as $Z_i / \cos \theta _i$ for TE and $Z_i \cos \theta _i$ for TM at a planar boundary^16^, leading to distinct angular reflection behaviors and the possibility of a Brewster angle (zero reflection) only for TM incidence. In reporting, reflection is treated as a *power* quantity; the air–skin value $R_0$ follows:

2 $$\begin{aligned} R_0 \;=\; \left| \Gamma _0\right| ^{2} \;=\; \left| \frac{Z_{\textrm{in,skin}}-Z_0}{Z_{\textrm{in,skin}}+Z_0}\right| ^{2}, \end{aligned}$$

with $Z_{\textrm{in,skin}}$ the input impedance seen into skin and $Z_0$ the free-space impedance. The overall transfer matrix is used to calculate the total power reflection at the air-skin interface. After accounting for forward and backward propagating waves, the electric field distribution *E*(*z*) within each layer is calculated. The SAR distribution can then be calculated as follows:

3 $$\begin{aligned} \textrm{SAR}_n(z) = \frac{\sigma _n |E_n(z)|^2}{2\rho _n}. \end{aligned}$$

A power-conservation audit is evaluated for each case:

4 $$\begin{aligned} \int _{0}^{L} \rho (z)\,\textrm{SAR}(z)\,dz \;+\; S_{\textrm{deep}} \;\approx \; \bigl (1 - R_0\bigr )\,S_{\textrm{inc}}\,\cos \theta , \end{aligned}$$

and simulations are accepted when the relative mismatch is below 5% for $\theta \le 70^\circ$. This energy-balance check provides a compact numerical validation of the multilayer solver and was found to be satisfied to within 1–3% for the vast majority of parameter combinations, with slightly larger deviations only at the most grazing angles. For additional verification, limiting cases at normal incidence were compared against classical Fresnel formulas and reported absorption factors for similar planar tissue models^8,9^, yielding consistent reflection and transmission coefficients.

### Pennes bioheat equation

The steady-state Pennes bioheat equation for tissue layer *n* is,

5 $$\begin{aligned} -\frac{\partial }{\partial z}\left( \kappa _n \frac{\partial T_n}{\partial z}\right) + \textrm{SAR}_n(z) + b_n\,[T_n(z) - T_b] + A_n = 0, \end{aligned}$$

where $\kappa _n$ is thermal conductivity, $b_n$ is the blood perfusion term, $T_b$ is blood temperature, and $A_n$ is metabolic heat generation, with boundary conditions

6 $$\begin{aligned} \kappa _1 \frac{\partial T}{\partial z}\bigg |_{z=0}&= h\,[T_1(0) - T_{\text {air}}], \end{aligned}$$

7 $$\begin{aligned} T_3(L)&= T_b, \end{aligned}$$

and interface continuity of temperature and heat flux. The Pennes formulation remains the most widely used macroscopic model for perfusion-mediated heat exchange in living tissues^22^, and it has been successfully employed in a wide range of RF and microwave heating studies^10,21,23^. In the implementation, the temperature rise is obtained directly from the steady-state $\Delta T$ equation:

8 $$\begin{aligned} -\,\frac{d}{dz}\!\left( \kappa (z)\,\frac{d\,\Delta T}{dz}\right) + b(z)\,\Delta T(z) \;=\; \rho (z)\,\textrm{SAR}(z), \end{aligned}$$

with Robin boundary $-\,\kappa (0)\Delta T'(0)=h\,\Delta T(0)$ and deep Dirichlet condition $\Delta T(L)=0$. Solving (8) avoids subtracting two absolute-temperature fields and improves numerical conditioning. The finite difference method (FDM) discretization uses central differences for the second derivative and forward/backward differences at boundaries. The resulting tridiagonal system is solved for the temperature distribution *T*(*z*) with and without SAR heating; the temperature elevation is expressed as,

9 $$\begin{aligned} \Delta T(z) = T_{\text {with SAR}}(z) - T_{\text {without SAR}}(z). \end{aligned}$$

For verification of boundary conditions and solver implementation, a published three-layer 60 GHz slab benchmark (forearm configuration) is reproduced in Supplementary Fig. S5^21^. The model validation is therefore based on three complementary elements: (i) analytical power-balance checks as in (4), (ii) reproduction of an external three-layer benchmark including both EM and thermal quantities^21^, and (iii) consistency with experimental and numerical data on RF absorption in layered tissues and fat channels reported in the literature^2,3,11,12,19,20^. A full transient solution of the Pennes equation, which is particularly important for short therapeutic exposures^23^, lies outside the scope of the present steady-state analysis but is acknowledged as an important direction for future work.

Notation for Eqs. (2)–(9)***:*** $\sigma$ electrical conductivity; $\rho$ mass density; *E* electric field; $\kappa$ thermal conductivity; *b* perfusion coefficient; $\Delta T$ steady-state temperature rise; *h* convective coefficient; $T_{\textrm{air}}$ ambient temperature; $T_b$ deep/core temperature; *L* total slab thickness; $R_0$ power reflection at the air–skin boundary; $Z_{\textrm{in,skin}}$ input impedance seen into skin; $Z_0$ free-space impedance; $S_{\textrm{inc}}$ incident plane-wave power density; $S_{\textrm{deep}}$ residual Poynting flux at depth; $\theta$ incidence angle.

## Results

The results presented here calculate power reflection, SAR distributions, and temperature elevations across the entire parameter space examined, showing how frequency, morphology, and exposure conditions influence each other. Unless otherwise noted, all reported temperature rises correspond to the steady-state solution of (8) under a fixed incident power density of 50 $\mathrm {W/m^2}$, which is comparable to or higher than the reference levels used in current exposure guidelines for localized assessments^4,5^.

### Reflection analysis across tissue interfaces

**Fig. 3:**
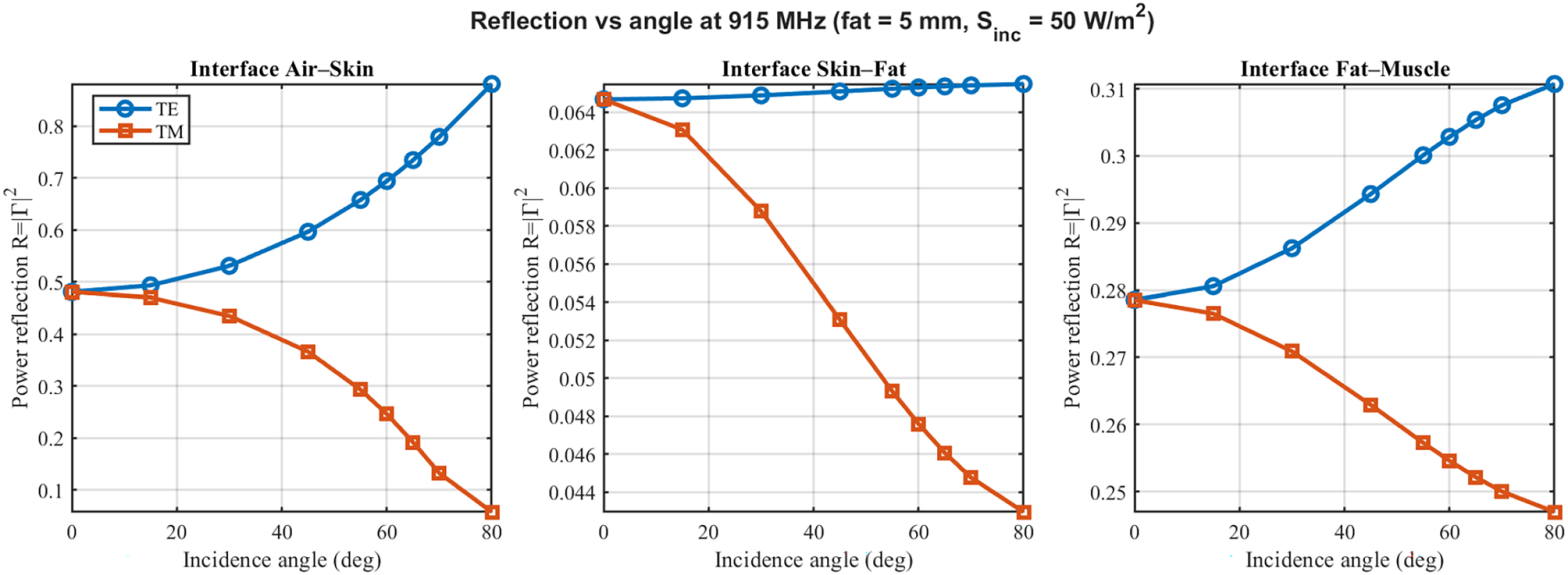
Consolidated angle-dependent power reflection ($R=|\Gamma |^2$) at 915 MHz. The panels show the air–skin (left), skin–fat (middle), and fat–muscle (right) interfaces side-by-side. Results are physically validated to strictly satisfy energy conservation ($R \le 1$). TE (blue circles) and TM (orange squares) polarizations diverge at oblique angles, with distinct Brewster-like minima visible for TM. (Representative fat thickness 5 mm; $S_{\textrm{inc}}=50$ W $\hbox {m}^{-2}$).

As shown in Fig. 3, power reflection at the air-skin, skin-fat, and fat-muscle interfaces is summarized for 915 MHz over angle for TE/TM at a representative fat thickness of 5 mm. TE and TM curves diverge at oblique angles due to polarization-dependent boundary impedances; pronounced Brewster-like minima are observed in TM where applicable. Values are clamped to $0\le R\le 1$ and checked by the budget in (4). Additional reflection panels at 433 MHz and 2.45 GHz are provided in Supplementary Figs. S1 and S3, respectively. The interface-specific reflection values in Fig. 3 represent the effective power reflected at each boundary within the multilayer system, and hence differ from simple two-medium Fresnel predictions based only on the local permittivities; multiple reflections, interference, and the TM Brewster condition at the skin–fat interface jointly shape the non-intuitive angular trends, in agreement with multilayer theory^8,16^.

### Temperature elevation and fat thickness effect

**Fig. 4:**
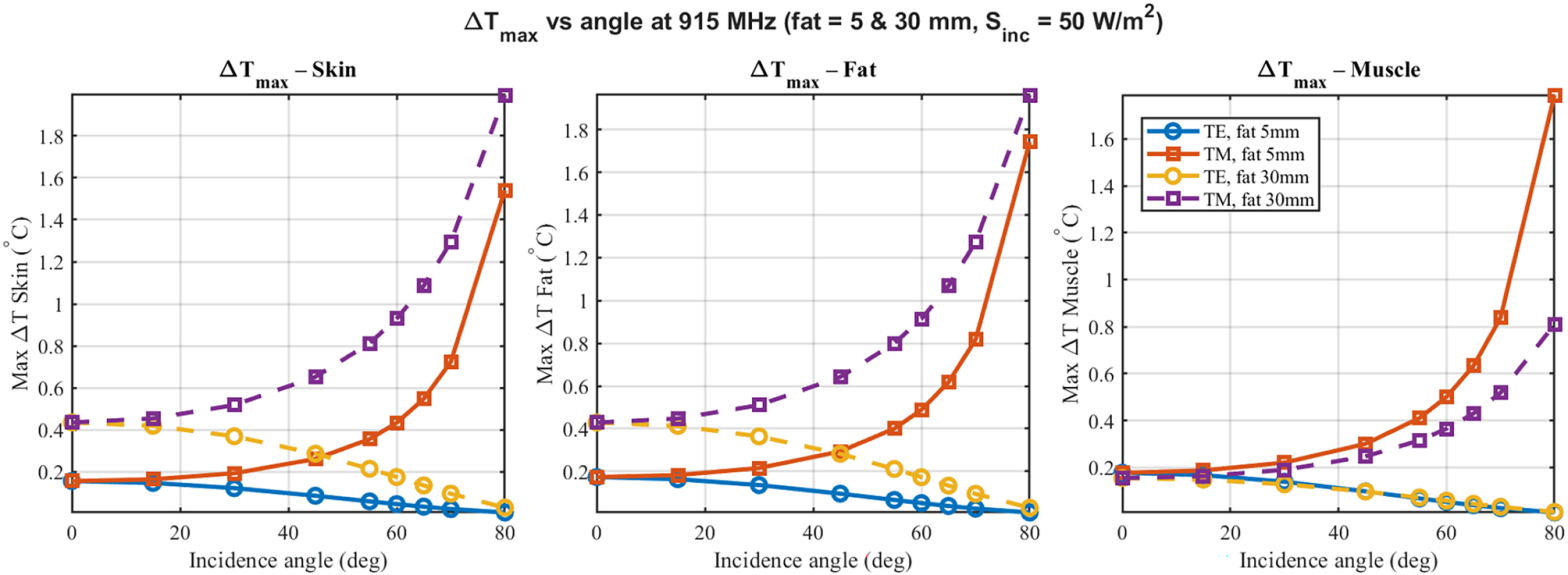
Maximum steady-state temperature rise ($\Delta T_{\max }$) at 915 MHz. Thermodynamically consistent positive heating ($\Delta T > 0$) is observed across skin (left), fat (middle), and muscle (right) layers. The plots compare thin (5 mm, solid lines) and thick (30 mm, dashed lines) fat morphologies for both TE and TM polarizations, highlighting the significant insulating effect of the fat layer.

**Fig. 5:**
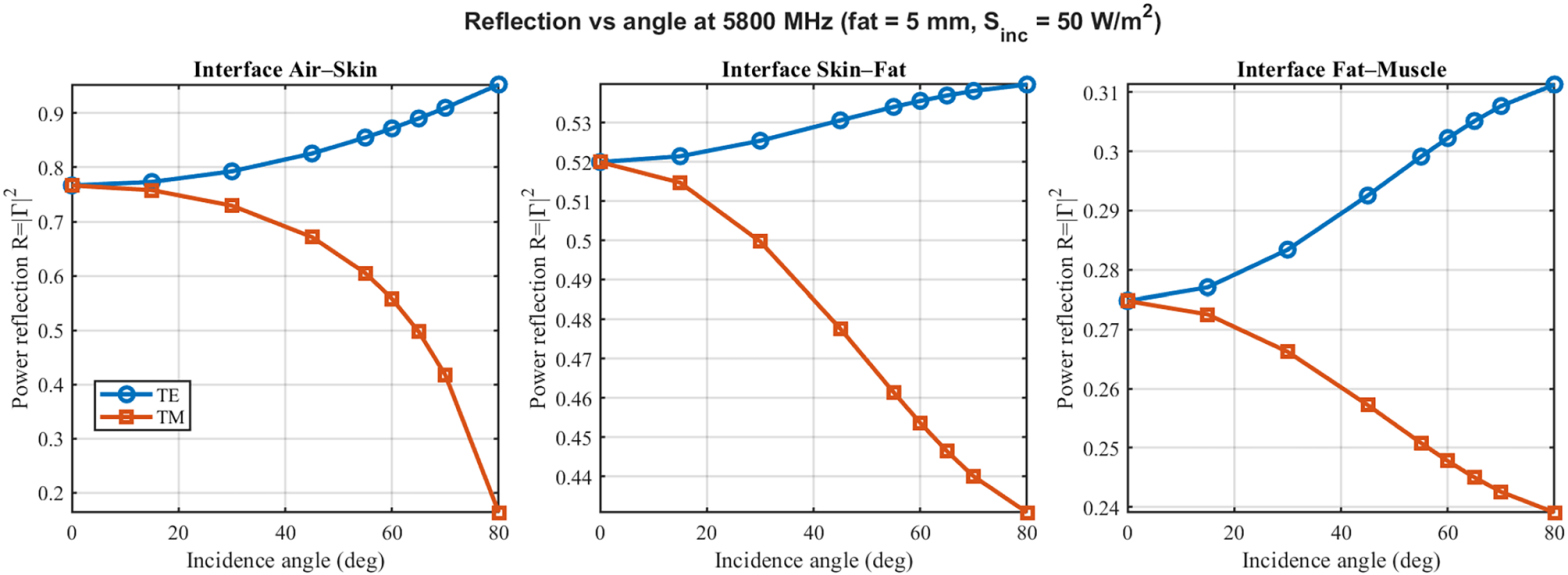
Consolidated power reflection at 5.8 GHz. Layout and conventions follow Fig. 3. The higher frequency results in modified impedance matching conditions, yet the model maintains strict adherence to power budget limits ($R \le 1$) across all incidence angles.

**Fig. 6:**
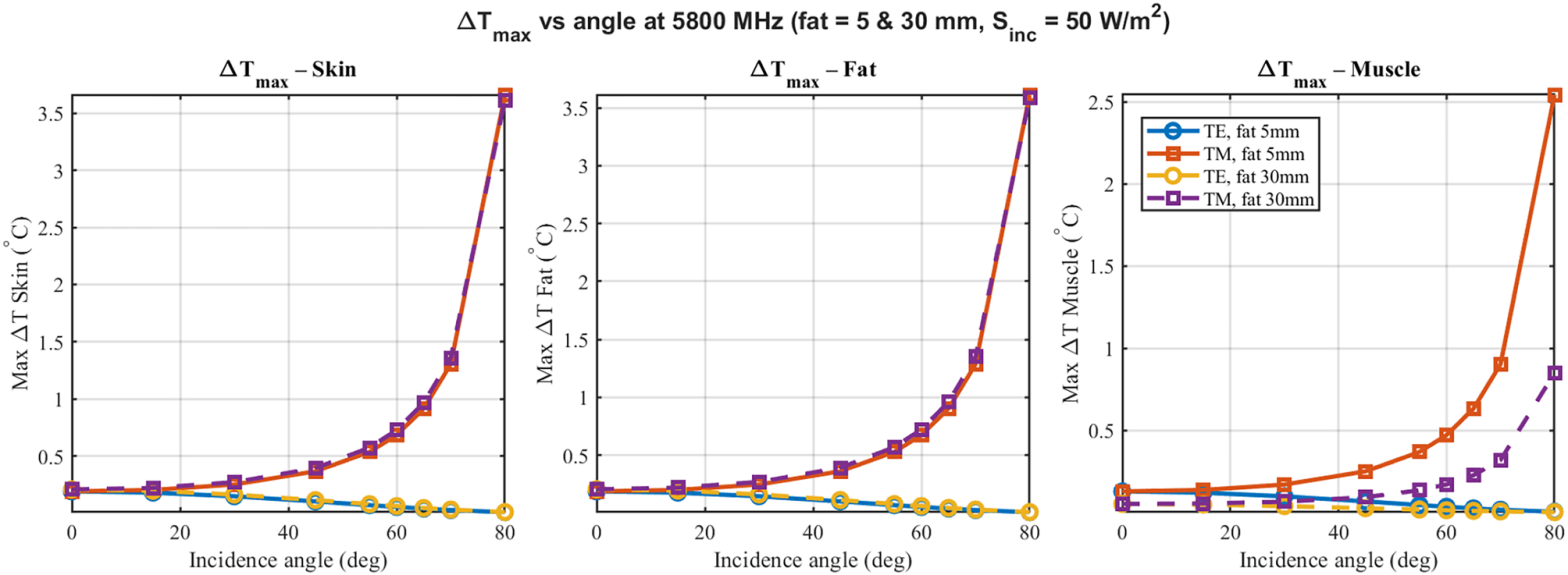
Maximum steady-state temperature rise at 5.8 GHz. Compared to 915 MHz, significant surface heating is observed in the skin layer due to the reduced penetration depth. The results confirm the solver’s ability to capture frequency-dependent heating profiles while ensuring positive temperature elevations.

Figure 4 shows $\Delta T_{\max }$ versus angle in skin, fat and muscle at 915 MHz for TE/TM and fat thicknesses 5 mm and 30 mm. $\Delta T$ is obtained from the direct steady-state Pennes form (8) with boundary conditions as stated below that equation; energy conservation holds within 5% by (4). The 5.8 GHz counterpart appears in Fig. 6. Supplementary Figs. S2 and S4 provide the corresponding 433 MHz and 2.45 GHz panels. At 915 MHz, temperature rises remain modest ($< 0.3^\circ$C) across all layers, driven by deeper field penetration (Fig. 4). However, at 5.8 GHz, the reduced penetration depth results in significantly higher energy deposition in the skin layer. As shown in Fig. 6, skin temperature rise reaches approximately 3.5$\vphantom{0}^\circ$C for TM polarization at grazing angles, highlighting a critical frequency-dependent thermal risk that is not apparent at lower ISM bands. Furthermore, the insulation effect of the thicker fat layer (30 mm) tends to increase the skin temperature rise compared to the thin fat case (5 mm). (Fig. 5)

### Comparative evaluation

Earlier studies of multilayers focused on normal incidence or fixed polarization. As shown here, once tissue layering is accounted for, reflection under realistic oblique exposure (0$\vphantom{0}^\circ$–80$\vphantom{0}^\circ$) remains moderate, and fat thickness dominates deeper heating. The present analysis reveals that the importance of incidence angle and polarization is highly frequency-dependent. At 433 and 915 MHz, tissue morphology (fat thickness) is the dominant factor governing steady-state temperature rise. However, at 5.8 GHz, the incidence angle and TE/TM polarization become critical: TM polarization at grazing angles leads to sharp increases in skin reflection and absorption, resulting in localized temperature spikes that are absent at lower frequencies. This observation is consistent with previous one-dimensional worst-case studies at RF frequencies, which also reported relatively small temperature differences between normal and oblique plane-wave incidence when averaged over macroscopic tissue volumes^10,21^. In contrast, studies at higher power at mmWave bands have shown that angle effects predominate^14,15^. Prior systematic reviews of radiofrequency wave exposure in confined environments identified the need for comprehensive assessment methodologies^26^, but most existing studies focus on either electromagnetic or thermal analyses in isolation, rarely combining reflection, absorption, and temperature assessment in an integrated manner. As compared to most radiofrequency wave exposure studies in confined environments, which are typically limited to the evaluation of electric field and SAR parameters, this study incorporated thermal analysis for a more comprehensive assessment of tissue heating under realistic exposure conditions. Moreover, the finding that fat can enhance transmission into deeper tissue layers for particular combinations of frequency and thickness aligns with experimental observations of adipose tissue acting as a relatively efficient propagation medium for intra-body communication links in the R- and ISM-bands^17,18,20^, thereby supporting the physical plausibility of the model predictions.

## Conclusion

By varying the thickness of fat layers, incident angles, and polarization characteristics of a layered tissue model across four ISM frequency bands, this research provides a detailed parametric analysis of electromagnetic wave interaction in a layered tissue model. The underlying physical mechanisms governing energy absorption could now be examined systematically via this analytical method. Based on the complex interplay among these factors, there are substantial differences in exposure metrics such as SAR and temperature elevation. Due to wave interference, the fat layer beneath the skin acts as an impedance transformer that changes with frequency, and specific fat thicknesses can actually increase power transmission into tissues. Consequently, morphological variations must be taken into account when conducting dosimetric studies. During angled incidence, the Brewster angle effect greatly changes the reflection characteristics of TM polarization when compared to TE polarization. For TE polarization, reflection decreases more gradually with angle and no true Brewster zero exists, so that TE incidence generally yields somewhat higher power reflection than TM at the same obliquity in the considered frequency range. It has also been observed that for TE polarization, oblique incidence can sometimes result in higher superficial SAR than normal incidence. The combined electromagnetic-thermal analysis of the study quantified the transition from deep-tissue heating to superficial heating at higher frequencies. Poor heat dissipation in the fat layer contributes to the increase in surface temperature. Specifically, at 5.8 GHz, skin temperature rises were found to be significantly higher than at lower ISM frequencies, confirming that the combination of shallow penetration depth and oblique TM incidence represents a worst-case exposure scenario. It is clear that the planar 1D model overlooks tissue heterogeneity and curvature, but the comprehensive parametric investigation still sheds light on fundamental wave processes and reveals the worst-case scenarios. Accordingly, the present results are best interpreted as a mechanistic baseline and as a tool for rapidly exploring the influence of frequency, polarization, angle, and morphology; for application to specific therapeutic or diagnostic devices, confirmation with subject-specific two- or three-dimensional full-wave simulations and, where feasible, with phantom or in vivo measurements remains essential. Future extensions could incorporate transient bioheat modeling, spatially varying perfusion, and more realistic geometries to further bridge the gap between analytical insight and clinical practice^23^.

## Supplementary Information

Supplementary Information.

## Data Availability

The datasets used and/or analysed during the current study available from the corresponding author on reasonable request.
