# Supplementary material for: Parametric analysis of electromagnetic wave interactions with layered biological tissues for varying frequency, polarization, and fat thickness
Source: Sci Rep. 2025 Dec 26;16:3445. doi: 10.1038/s41598-025-33460-2 (PMC12835268; doi:10.1038/s41598-025-33460-2)
Supplement: Supplementary file 1 — Supplementary Information. [file 41598_2025_33460_MOESM1_ESM.pdf]

Supplementary Information

The supplementary figures are reproduced here for completeness and are referenced in the main text where appropriate (not in the Abstract). All panels use the same plotting style as the main figures: TE (circles), TM (squares), fat thickness indicated in the legend, and $S_{inc} = 50\ \mathrm{W\,m^{-2}}$ unless otherwise specified.

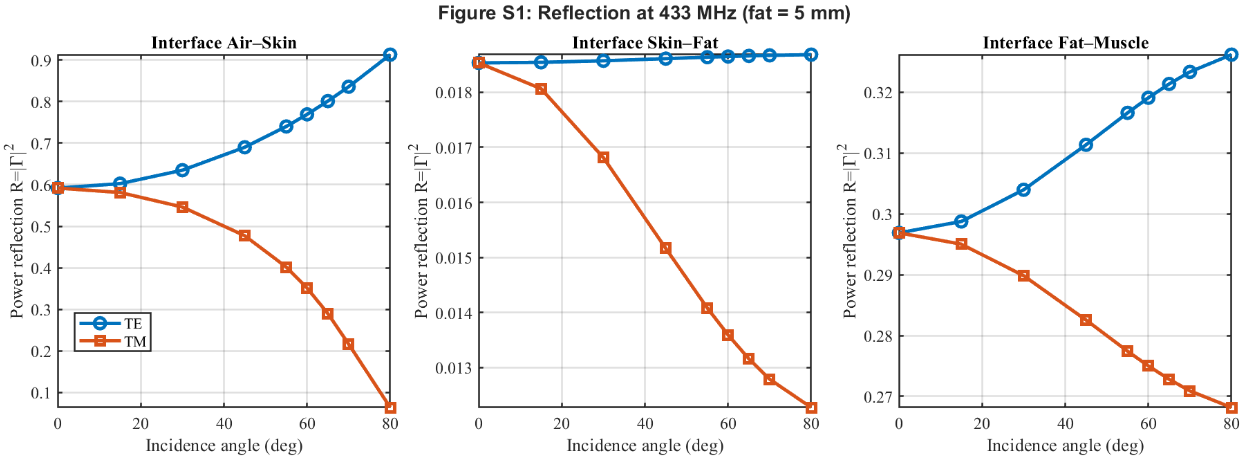

Figure S1. Figure S1: Consolidated power reflection at 433 MHz. Angle-dependent *power* reflection $R = |\Gamma|^2$ at (left) air–skin, (middle) skin–fat, (right) fat–muscle for TE/TM; representative fat thickness 5 mm.

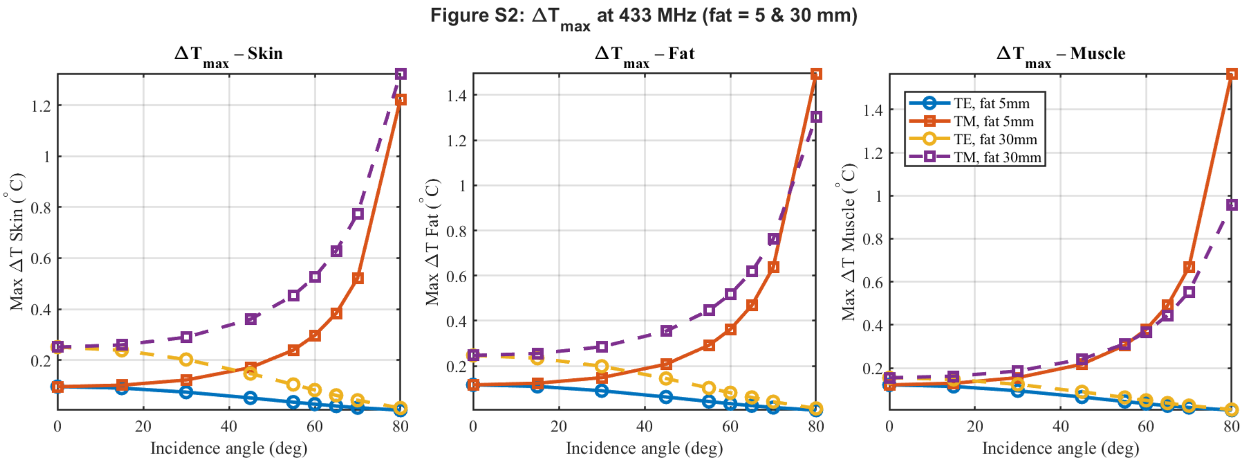

Figure S2. Maximum steady-state temperature rise at 433 MHz. ΔT_{max} vs angle in (left) skin, (middle) fat, (right) muscle for TE/TM; fat thicknesses 5 mm (solid) and 30 mm (dashed).

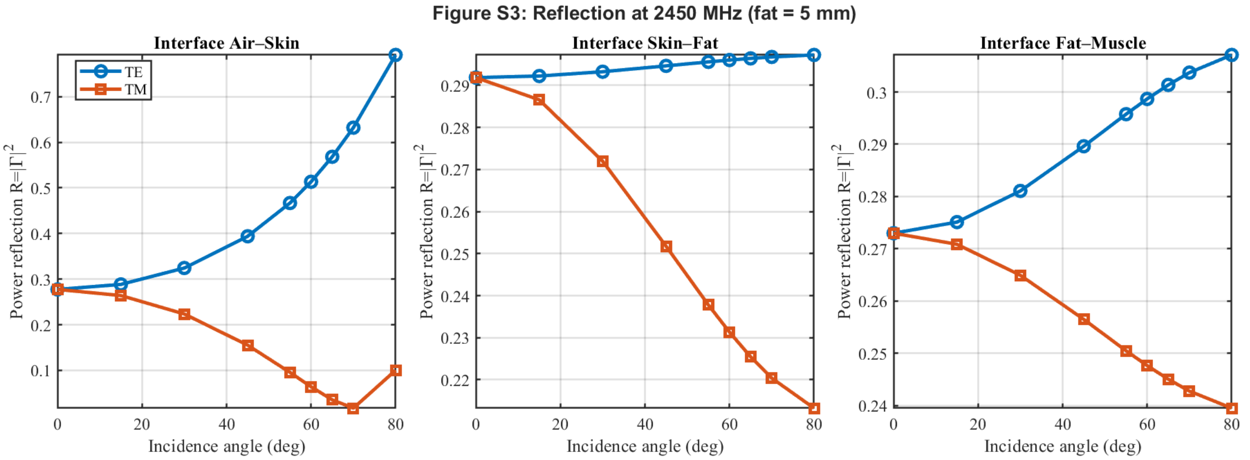

Figure S3. Consolidated power reflection at 2.45 GHz. Angle-dependent *power* reflection $R = |\Gamma|^2$ at (left) air–skin, (middle) skin–fat, (right) fat–muscle for TE/TM; representative fat thickness 5 mm.

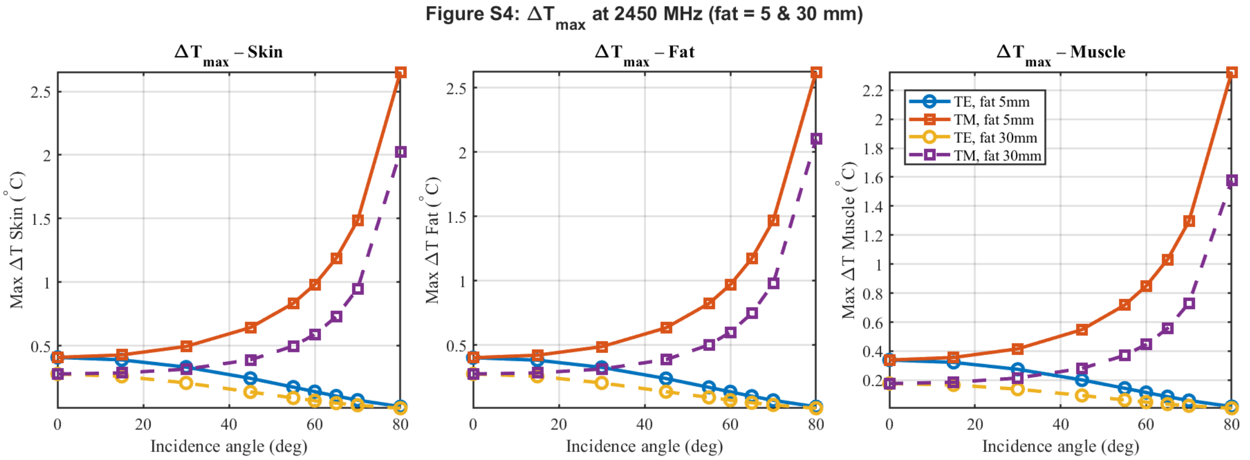

Figure S4. Maximum steady-state temperature rise at 2.45 GHz. ΔT_{max} vs angle in (left) skin, (middle) fat, (right) muscle for TE/TM; fat thicknesses 5 mm (solid) and 30 mm (dashed).

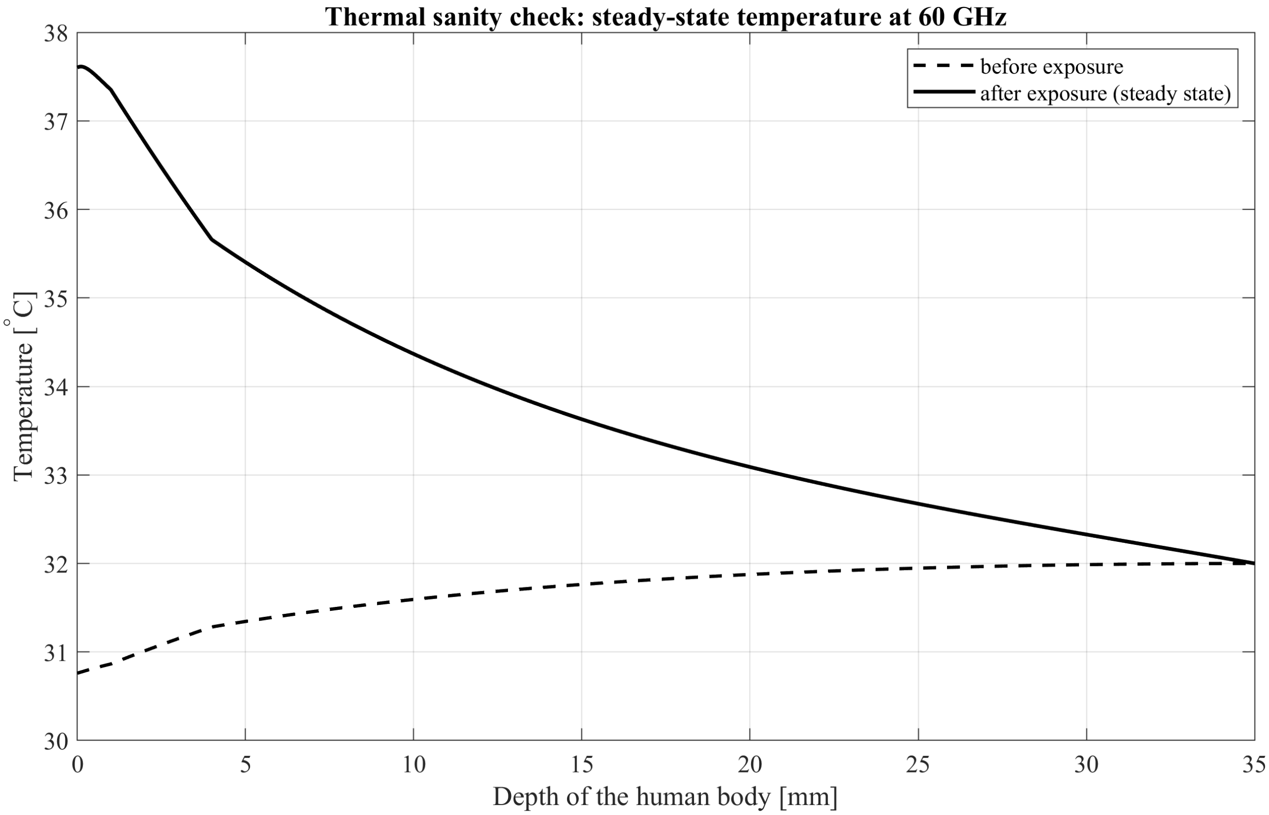

Figure S5. External solver check at 60 GHz. Three-layer slab (1 mm skin / 3 mm fat / 31 mm muscle), normal incidence; $S_{\text{inc}} = 500\ \text{W m}^{-2}$, $h = 7\ \text{W m}^{-2}\,{}^{\circ}\text{C}^{-1}$, $T_{\text{air}} = 23.6^{\circ}\text{C}$, $T_b = 32^{\circ}\text{C}$. Dashed: baseline (no RF heating); solid: steady state under exposure. Included solely to verify boundary conditions and the steady-state thermal solver; it is not part of the ISM-band parametric results.
